# Children staying in hospital: a research on psychological stress of caregivers

**DOI:** 10.1186/1824-7288-36-40

**Published:** 2010-05-25

**Authors:** Elena Commodari

**Affiliations:** 1 Department of Educational Processes, Faculty of Education, University of Catania, Via Ofelia, Catania 95100, Italy

## Abstract

**Background:**

Having a child hospitalized is a stressful event for parents. Previous studies have found increased stress in families with children affected by different kinds of pathologies, and analyzed disease related objective variables producing stress. However, most of these studies recruited caregivers of children with chronic or serious illnesses, and focused on evaluation of objective environmental stressors and did not consider subjective "perception" of stress. The aim of this study was to investigate perception of acute stress in caregivers taking care of children without serious physical damage that were hospitalized for short periods. Moreover, some variables, such as recreational and school services offered to children, influencing perception of cognitive, physiological and behavioral state relating to the sensation of "being stressed" were analyzed.

**Methods:**

This study was realized with a sample of caregivers of children hospitalized for mild acute diseases. Research was conducted using two standardized tests, PSM (Psychological Stress Measure) and STAI (State Trait Anxiety Inventory), whose characteristics of reliability and validity had been successfully established.

**Results:**

Present data showed that caregivers of hospitalized children perceived high levels of stress and anxiety. Perception of stress was influenced by the degree of kindred with patients, length of hospitalization, and, notably, participation in some of the activities offered to children, mainly school services.

**Discussion:**

Findings showed that child hospitalization is a stressful event for caregivers, even if hospitalization is for middle and transient pathologies. Perception of stress was influenced by length of hospitalization, and by degree of kindred. Findings even suggest that some services offered to children can modulate caregivers' perception of stress and impact of hospitalization. Caregivers whose children used school services describe themselves as less irritable and with higher emotional control compared to other caregivers. Considering the importance of education in a child's life, the possibility to continue school activities helped caregivers to feel less under pressure. In the light of this finding, amelioration of scholastic activities in pediatric departments may represent a critical point in order to provide a more agreeable hospital stay for children and their caregivers and, as a consequence, improve family involvement in care management.

## Background

Parents have an important role in the promotion of their children's health, being the primary agents involved in direct care, providing access to health services and modeling attitudes and behaviors that influence children's wellbeing [1]. Parents' psychosocial functioning is important for children's physical and mental health outcomes, and their attitudes during a child's illness, especially during hospitalization, may deeply influence the child's adherence to the care and impact of the disease.

Having a child hospitalized is a stressful event for parents who often experience anxiety and depression during the period of hospitilization. According to classical definition [2], stress is a non-specific response of the body to any excessive environmental request. The reaction to stress is not directly related to the exposure to stressors but is mediated by the individual emotional response. Stress is, in fact, a process embracing several components including stressors, defined as events that pose a challenge to the subject, psychosocial mediators, constructs that enable the subject to evaluate the nature of the situation, and the stress response, typically a measure of the emotional reaction elicited in response to the stressor [3,4]. Moreover, a stress response may often include anxiety as an incorporate component. If stress is understood as a critical event, anxiety follows this event (e.g., in the form of a post-traumatic disorder); if stress is defined as a response to such event, anxiety is part of the response pattern. Whereas if stress is defined as a transactional encounter between a person and a situation, anxiety is an accompanying emotion of stressful encounters [4]. Empirical and theoretical inquiry has led to a distinction between minor stressors, such as daily hassles, and major challenges such as life events including hospitalization [5]. Research has also demonstrated that several psychosocial mediators are critical determinants of the stress response; for instance, inadequate social support has been isolated as a significant predictor of stress in patients with HIV infection [6], women with postpartum depression [7] and patients with cancer [8,9].

According to the complexity of the stress phenomenon, several models of stress response have been developed. Among them, the bio-psycho-social model describes psychological stress as the state of normal tension, preoccupation and agitation reported by many people, defining it as the relationship between the person and the environment that is appraised by the person as taxing or exceeding his or her resources and endangering his or her well being [10]. Developed outside the field of psychopathology, the bio-psycho-social model is useful for assessing psychological stress in primary care and population health research. The bio-psycho-social model of stress includes environmental parameters and individual processes of perception and coping with stressors. The effects of stress are, in fact, directly linked to coping. The classical Lazarus theory [11,12] defines coping as constantly changing cognitive and behavioral efforts to manage specific external and/or internal demands that are appraised as taxing or exceeding the resources of the person. Managing can include minimizing, avoiding, tolerating and accepting the stressful condition, as well as attempts to master the environment.

According to the bio-psycho-social model, in some circumstances stress can act as a precipitating factor in the development of various physical and mental disorders [10,13,14] and result in physical or mental illness. Stress-induced disorders occur only as a result of stress when it is of great intensity or long duration or when other pathogenic processes are also present. Thus, psychological stress is different from psychopathological diseases, which are dysfunctional and morbid, and refers to a set of affective, cognitive, somatic and behavioral manifestations within the range of functional integrity.

The impact of pediatric illnesses and disabilities on family stress has been well documented in the literature. Several studies have found increased stress in families of children with various diseases such as asthma, diabetes, heart disease and other chronic pathologies [15-19]. Evidence brought forward suggests that parents' stress has important implications for the child's health and behavioral outcomes [20,21]. Parental stress is influenced by particular conditions, such as characteristics of family structure. For instance, adoptive mothers perceived significantly higher levels of stress during their child's hospitalization compared to mothers whose biological children were hospitalized [22]. Other researches have led to the identification of several psychosocial mediators that have been found to be important determinants of the stress response across a variety of settings and populations [23,24]. Intensive psychosocial intervention in inpatients and outpatients is found to have a positive impact on child and family functioning, therefore reducing stress levels [23]. Nurses, too can play a central role in these interventions by helping families to identify and mobilize resources in medical and community settings [24].

The purposes of this study were: firstly, to deeply analyze the global perception of stress and its related cognitive, physiological and behavioral aspects in a sample of caregivers of hospitalized children. Unlike most of the researches on stress responses which have studied objective variables producing stress or psychological damage connected to stress, the present research investigated subjective "perception" of stress, with the aim of evaluating the individual's feelings of "being under pressure". In particular, given the fact that psychological stress is different from psychopathological diseases [10,14], the present study is focused on the evaluation of psychological stress as a set of affective, cognitive, somatic and behavioral manifestations within the range of functional integrity. Moreover, while most of the previous studies involved caregivers of patients with chronic and serious illnesses [14,16], this study recruited caregivers of pediatric patients with middle and transient pathologies with the purpose of analyzing psychological reactions related to a particular situation such as hospitalization is by itself. The second purpose was to investigate whether variables such as recreational and school activities arranged for hospitalized children, contribute to influence emotional changes related to stress.

## Methods

### Setting and sample

This study was realized on a sample of hospitalized children's caregivers. Research involved the pediatric departments in all four public hospitals of a large Italian town. Departments admitted patients with similar pathologies, and had similar organization of physician and trained nursing staff.

In each department, only caregivers taking care of children diagnosed by a physician as affected by middle and transient pathologies were recruited. 55% of patients were affected by respiratory diseases, 30% by gastro-intestinal pathologies, 9% by allergic reactions and 11% by other transient pathologies. Caregivers attended children during the night and for almost 6 hours during the day.

**Figure 1:**
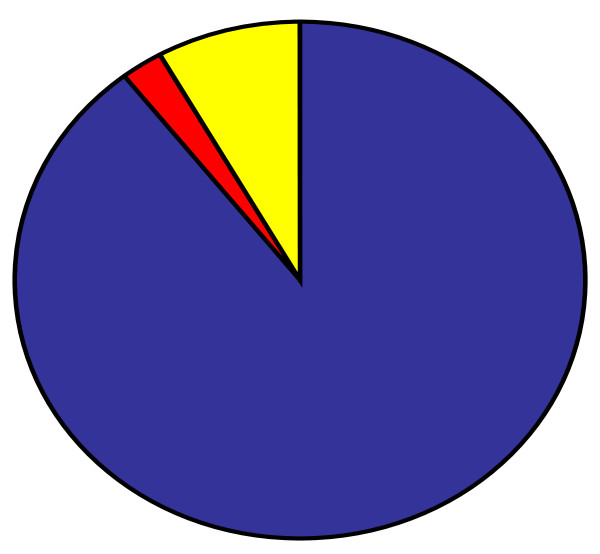
**Relatioship between caregivers and children in the recruited sample of hospitalized children**. Blue: parents, red: brother/sister, yellow: other relatives.

Research involved 219 caregivers (19 men and 200 women - mean age 32 years standard deviation: 8.71).179 caregivers were the mothers of children staying in hospital, 18 were the fathers, 5 were brothers or sisters, 17 were other relatives (grandmothers or aunts) (see figure 1).

As regards education, 7.9% of caregivers had a primary school level, 47.9% had junior high school level, 32.4% had a senior high school level, 12.3% had a degree.

All pediatric departments in which research was conducted supplied recreational and scholastic activities for the children. In each department there were voluntary groups offering entertainment activities for patients, toys were put at children's disposal and state-recognized teachers were available during school term time. Teachers were present in the morning while voluntary groups were generally present for a number of hours in the afternoon, several days a week. State-recognized teachers in the pediatric department guaranteed the children the right to study, according to the Italian laws on compulsory education. Recreational services were run by trained volunteers and managed by National Voluntary Services, working nationwide. Volunteers were not part of hospital staff, and made use of toys and material present in pediatric departments. Voluntary groups working in the hospitals had similar social statutes and organizations.

Hospitalized children were between < 1 year and 14 years old (mean age 4 years, sd 3.55).

The study was approved by the Ethic committee of each hospital and all subjects gave their written informed consent.

### Instruments

Research was conduct using two standardized tests, PSM - Psychological Stress Measure- [11], and STAI - State Trait Anxiety Inventory [25-27] , measuring perception of stress and level of anxiety, whose characteristics of reliability and validity had been successfully established. Caregivers also completed a multiple-choice questionnaire aimed at investigating some variables related to knowledge, use and appreciation of recreational, play and school activities arranged in each pediatric department, and supplied some personal details.

PSM [10,13,14] was used to measure stress. The PSM measures the stress intended as the result of a complex response system, rather than the result of a specific stressing circumstance. According to authors, response to stress is an attempt to handle tension manifesting itself in different psychological and physiological ways, as well as in differing degrees. Therefore, it is not a purely clinical response, or a symptom of incapacity, unless it becomes chronic. As a consequence, PSM was developed for the general population, with the purpose of overcoming limits related to use of clinical and psychiatric measurement tools. Psychiatric measurement tools were designed for pathologic disorders and validated using dysfunctional clinical populations, and were not particularly sensitive in non-clinical population.

PSM does not aim to test level of stress through an inferential method but rather focuses on the individual's state stress and makes references to people's experiences [18]. The questionnaire consists of 49 items, based on the various individual perceptions of cognitive, physiological, and behavioral state of subjects. PSM provides a global score of stress and some partial sub-scores: "loss of control/irritability", "psycho-physiological changes", "mental confusion/mental effort", "depressive anxiety", "pain or physical disturbances", "hyperactivity". Partial sub-scores regard the six principal clusters obtained by Di Nuovo, Rispoli, Genta, authors of the Italian translation of PSM [13], through a cluster analysis using single linkage-nearest neighbor. These clusters were comparable to the four categories (behavioural, somatic, cognitive, affective) used by Lemyre, Tessier and Fillon to individuate and select items during questionnaire construction. The global score and partial scores were automatically obtained through a computerized scoring system. PSM has emerged as a psychometrically sound procedure to measure perception of stress (Internal consistency - alpha Cronbach coefficient: .95; test-retest stability between .68 - .80; PSM normality of distribution: z = 94, p > 3). The PSM has been translated into different languages (i.e. English, Japanese, Italian) and these versions maintain the same heuristic statistics, normality of distribution and responsiveness of original version [14].

STAI [25-27] is a self-report assessment which includes separate measures of state and trait anxiety. Anxiety-state reflects a transitory emotional state and a condition of the human organism that is characterized by subjective, consciously perceived feelings of tension and apprehension, and heightened autonomic nervous system activity. Anxiety-state may fluctuate over time and can vary in intensity. In contrast, anxiety-trait denotes relatively stable individual differences in anxiety proneness and refers to a general tendency to respond with anxiety to perceived threats in the environment. STAI has emerged as a psychometrically sound procedure to measure anxiety (coefficient of reliability: .93). STAI scores have a direct interpretation: high scores on their respective scales mean more trait or state anxiety and low scores mean less.

## Results

Data analyses used descriptive statistics, analysis of variance and t test for independent sample, to detect group differences on dependent variables (PSM total and partial scores, STAI scores).

Data analysis showed high levels of stress and anxiety-state in caregivers. Perception of stress and level of anxiety-state were significantly higher compared to the normative sample (PSM, our sample mean 103.68, std deviation: 26.62; Italian norms mean 90.47; std deviation: 22.96; *t *= 39.192, p < .001; STAI state: our sample mean 54.33, std deviation: 11.69, Italian norms mean 42.68, std deviation: 11.19, *t *= 12.009, p < .001). As expected, the anxiety trait, that is a stable characteristic of personality, did not differ between our sample and normative sample; anxiety-trait scores have not been considered in the following analysis. Table 1 presents means and std deviation of all sample in PSM (global scores and partial scores) and in STAI.

**Table 1:** Mean and Standard Deviation in PSM and STAI

	Mean	Std. Deviation
PSM	103.68	26.652

Loss of control/irritability	1.77	.424

Psycho-physiological change	1.75	.435

Sense of effort/confusion	1.74	.437

Depressive anxiety	1.69	.462

Physical disturbances	1.56	.498

Hyperactivity	1.86	.345

STAI state	54.33	11.698

STAI trait	45.24	11.417

To evaluate if length of hospitalization influenced the perception of stress and the level of anxiety, the sample was differentiated into four groups in respect of the variable "days of hospitalization" (group 1:1-5 days; group 2: 6-10 days, group 3:11-15 days; group 4: 16 or more days). This differentiation was based on physician staff indications, regarding the more frequent range of lengths of hospitalization in departments. 172 children were hospitalized for 1-5 days, 35 for 6-10 days, 8 for 11-15 days and 4 for a period longer than 16 days. This distribution was expected, considering that inclusion criteria was to recruit only caregivers taking care of children with transient and non serious illnesses.

ANOVA showed significant differences in caregivers' perception of stress but not in anxiety (Table 2). Post hoc with Bonferroni correction highlighted that hospitalizations prolonged over 16 days produced a significant increase in stress compared to shorter hospitalizations.

**Table 2:** Mean and std deviation of PSM global scores and STAI scores differentiated respect variable "days of hospitalization"

	Hospitalization	Mean	Sd	F	Sig.
PSM	Group 1 1-5 days	101.99	25.21	3.06	.02*

	Group 2 6-10 days	105.80	31,89		

	Group 3 11-15 days	113.12	25.40		

	Group 4 16 or more	139.00	14.67		
STAI	Group 1 1-5 days	53.40	11.26	2.32	.07

	Group 2 6-10 days	57.43	13.07		

	Group 3 11-15 Days	58.33	11.698		

	Group 4 16 or more	64.75	10.30		

* *t *is significant with p < .05

Analysis of PSM partial scores showed that "days of hospitalization" influence "depressive anxiety" (F: 5.02, p = .002), "physical disturbances" (F = 3.16, p = .02), and "hyperactivity" (F = 2.70, p = .046) scores, that were highest in longer hospitalization.

Analysis respect variable "age of patients" showed that child age did not influence global score of stress and level of anxiety, even if analysis of variance on PSM partial scores revealed significant differences in "psycho-physiological changes". Indeed, PSM psycho-physiological changes scores were higher in caregivers attending younger children (F = 3,083, p = ,017).

Relationship between caregiver and child was, instead, a significant variable. Parents, brothers and sisters of a child staying in hospital perceived themselves more stressed and vulnerable compared to other relatives taking care of a child. Particularly, parents, brothers and sisters presented higher global scores of stress and higher scores in "loss of control" and "psycho-physical change" items (F = 3.094, p = .047; F = 3.181, p = .044) compared to other relatives. Interestingly there were no significant differences in anxiety-state (Table 3).

**Table 3:** Mean and standard deviation of PSM global and STAI state scores differentiated respect variable "relationship with patient"

		Mean	Std. Deviation	F	Sig.
PSM Tot	Parents	133.00	38,.80	2243.45	.042*

	Brother/Sister	110.22	32,.08		

	Other relatives	102.22	25,08		

STAI state	Parents	54.75	11.59	365.28	.069

	Brother/Sister	59.50	15.50		

	Other relatives	48.61	10.971		

Partial scores					
Loss of control/Irritability	Parents	1.78	418	3.094	.047*

	Brother/Sister	1.78	.428		

	Other relatives	1.25	.500		

Psycho-physiological Change	Parents	1.77	.500	3.181	.044*

	Brother/Sister	1.67	.424		

	Other relatives	1.25	.485		

Sense of effort/confusion	Parents	1.76	.427	3.047	.050

	Brother/Sister	1.67	.484		

	Other relatives	1.26	.500		

Depressive anxiety	Parents	1.70	.459	.402	.670

	Brother/Sister	1.50	.577		

	Other relatives	1.67	.485		

Physical disturbances	Parents	1.56	.497	.775	.462

	Brother/Sister	1.25	.500		

	Other relatives	1.56	.511		

Hyperactivity	Parents	1.87	.334	.849	.429

	Brother/Sister	1.75	.500		

	Other relatives		.428		

*t is significant is p < .05

Scores in stress and anxiety tests did not significantly differ with regard to the caregivers' age or level of education.

As previously described, all pediatric departments in which research was carried out had a play room for children and offered recreational and school activities which were managed by volunteers and state-recognized teachers. We also analyzed whether use of these services influenced perception of stress and anxiety.

Data analysis showed that most caregivers (93.2%) knew of the availability of the play room, even if only 50.2% of caregivers affirmed their children used them. 21.5% of children using the play room frequented it occasionally, the others once or twice a day. *t *test did not show any significant differences in caregiver stress and anxiety regarding the use of the play room.

As regards the presence of volunteers, most of the caregivers (92.2%) knew that volunteers to play with patients were periodically present, but only 50.2% affirmed their children used this service. Caregivers using volunteers' services obtained global score of stress and anxiety similar to those who did not. It is interesting that caregivers said that their children used the playroom mainly to participate in recreational activities. Probably children went into the playroom only when volunteers were present. All caregivers whose children used recreational services affirmed they were satisfied with the service, even if appreciation and use of recreational services did not influence perception of stress and anxiety.

As regards school service, 45.2% (n = 99) of children used this. 32.4% caregivers confirmed their children used the school service regularly, 12.8% occasionally. 97% of caregivers using school service were satisfied with the activities conducted by teachers. Interestingly, considering the age of the sample, only 34.2% of patients using school service were of school age. This data showed that also preschool children participated in teachers' activities; most of the children who did not use teacher services were, in fact, under the age of one.

Of note, caregivers of patients using school services obtained significantly lower scores in "loss of control/irritability" (t = 2.95, p = .003) compared to those that did not (Table 4)

**Table 4:** Mean and std deviation of PSM global and partial scores differentiated respect variable "use of scholastic service"

	Use of scholastic service	Mean	Std. Deviation	t	Sig
PSM total score	Yes	100.55	26.241	-1.589	.114
	
	No	106.28	26.818		

STAI	Yes	53.04	11.377	-1.490	.138
	
	No	55.40	11.898		
*PSM partial scores*					
Loss of control/Irritability	Yes	1.86	.350	2.953	.003*
	
	No	1.69	.464		

Psycho-physiological change	Yes	1.74	.442	-.354	.723
	
	No	1.76	.430		

Sense of effort/confusion	Yes	1.80	.404	1.657	.09
	
	No	1.66	.460		

Depressive anxiety	Yes	1.76	.431	1.859	.064
	
	No	1.64	.482		

Physical disturbances	Yes	1.58	.497	.503	.615
	
	No	1.54	.500		

Hyperactivity	Yes	1.88	.328	.614	.540
	
	No	1.85	.359		

## Discussion

This study showed that caregivers of children hospitalized for mild acute diseases perceived higher level of stress and anxiety compared to the control population.

The aim of this study was to investigate perception of acute stress in caregivers taking care of children with no significant physical problem, not suffering from chronic or disabling disturbances, and hospitalized for short periods only. The high level of perceived stress and anxiety shown in our sample was, therefore, strictly due to the specific "condition" of hospitalization itself, rather than from the seriousness of illness. Stress is, in fact, associated with unpleasant experiences producing tension, and hospitalization is itself a stressful situation.

Stress increased significantly with the addition of days of hospitalization and was higher in parents, brothers and sisters of the child in hospital compared to other relatives, regardless of the age of the patient.

Parents, brothers and sisters also experienced sensations of loss of control and confusion, and presented physical disturbances, such as digestive disturbances or pain, more frequently than other relatives. Probably emotional involvement is greater in the next of kin compared to other relatives taking care of children staying in hospital.

Interestingly, the anxiety state in caregivers did not differ as regards the length of hospitalization or degree of kindred with patient. These data confirmed the complexity of stress reaction, as a global response of the organism, respect anxiety-state, referring to a transitory state and feeling of apprehension [24-26] , that may be associated to psychological stress. Presumably, hospitalization is a condition which increases both stress and anxiety levels, but perception of stress is more directly influenced by specific modulation of stressful situations and by the emotional and cognitive involvement of caregiver. These data tally with the bio-psycho-social model of stress and with the Lazarus coping theory [10-12], according to which appraisal influences stress response.

Participation in recreational activities conducted by volunteers did not reduce the sense of pressure in caregivers. On the other hand, caregivers whose children used school services describe themselves as less irritable and with higher emotional control compared to other caregiver.

Considering the importance of education in a child's life, the opportunity to continue scholastic activities aided caregivers in feeling less pressurized themselves. Probably, the chance to send their children to school, even in hospital, gave a sense of continuity to daily experience, inevitably interrupted by hospitalization, even if brief. School in hospital is a guarantee of the child's right to study, and teachers' activities can be considered not only as a means of alleviating temporary forced isolation but also as a way that will favor a more rapid reintegration into daily scholastic context after the child is discharged from hospital.

Caregivers perceived state-recognized teachers in a different way to volunteers: teachers, with their activities, contributed to reducing confusion and irritability. This did not happen with volunteers' work. These data might encourage the need to consider a clearer definition of the role of volunteers in hospitals and different management of services supplied, with the aim of increasing trust in caregivers, so that recreational and play services could really contribute to ameliorate adjustment to hospitalization both in children and caregivers. In fact, it has been shown that reduction of stress in caregivers of hospitalized children contributes to establishing more effective treatment planning and family involvement in care management [23,24]. Moreover, positive emotional responses and cognitive attitudes of caregivers increase adherence to care and a milder perception of illness in the child.

In conclusion, these issues highlighted the need to strengthen school services in hospitals, offering all children, including bedridden children, the possibility to continue the school curricula, and they also underlined the need to improve the research into family involvement in Italian pediatric hospitals.

## Competing interests

The authors declare that they have no competing interests.
